# Female Affiliation and Status in Semi‐Free‐Ranging Chimpanzees

**DOI:** 10.1002/ajpa.70244

**Published:** 2026-04-08

**Authors:** Margaret A. H. Bryer, Paige Barnes, Kris H. Sabbi, Skylar Brodnan, Charlie MacKenzie, Isabelle Monroe, Natalia Camargo Peña, Phillip Sekulya, Innocent Ampeire, Amos Okello, Stanley Kyama, Boris Waiga, Hillary Aligumisiriza, Joseph Masereka, Joseph Kaale, Titus Mukungu, Joshua Rukundo, Zarin P. Machanda, Alexandra G. Rosati

**Affiliations:** ^1^ Department of Psychology University of Michigan Ann Arbor Michigan USA; ^2^ Department of Anthropology University of Wisconsin Madison Wisconsin USA; ^3^ Department of Human Evolutionary Biology Harvard University Cambridge Massachusetts USA; ^4^ Ngamba Island Chimpanzee Sanctuary/Chimpanzee Trust Entebbe Uganda; ^5^ Department of Anthropology and Biology Tufts University Medford Massachusetts USA; ^6^ Department of Anthropology University of Michigan Ann Arbor Michigan USA

**Keywords:** affiliation, dominance, primates, sex differences, social behavior

## Abstract

**Objectives:**

Sex differences in social behavior and status are pervasive across primates and other mammals. In the wild, chimpanzees ( *Pan troglodytes* ) exhibit many sex‐typed behaviors: adult female chimpanzees exhibit lower aggression, are subordinate to adult males, and also are generally less social than males. This pattern is thought to partly reflect the energetic constraints (e.g., feeding competition) that wild female chimpanzees face.

**Materials and Methods:**

We test the drivers of sex differences in chimpanzee behavior by examining a semi‐free‐ranging African chimpanzee sanctuary group where individuals are provisioned, an environment that should relax ecological constraints on socializing. Using two years of focal observations on a group with 45 chimpanzees (17 males, 28 females), we examined sex differences in social proximity, grooming, aggression, coalition formation, and dominance.

**Results:**

In contrast to patterns in wild chimpanzees, we found that males and females in this population exhibited comparable rates of affiliative behavior. Males engaged in more aggressive behavior overall than females, as in the wild. However, females were equally likely to aggress either sex, and a high proportion of female aggression involved coalitions. Finally, David's scores revealed that a few of the adult females outranked some of the lower‐status adult males.

**Discussion:**

These findings show that sex differences in chimpanzee social behavior are partially flexible, and females may show more affiliation, cooperation, and higher status when ecological conditions are favorable. More generally, some forms of female power can emerge even in a species with strong sex biases in behavior for male affiliation and status.

## 1 Introduction

Understanding the drivers of sex differences in behavior, and the contexts that may promote social power in females versus males, are key questions in biology (Clutton‐Brock and Huchard 2013a; Davidian et al. 2022; Huchard et al. 2025; Lewis 2018). Across primates and other mammalian taxa, males are often larger, more aggressive, and have higher dominance status than females (Clutton‐Brock 2007; Dixson 1998; Plavcan 2001). A primary explanation for these sex differences is sexual selection: since reproduction is more energetically costly for females, and male mammals often provide little or no parental care, males experience greater reproductive skew and more intense intrasexual competition (Andersson 1994; Clutton‐Brock 2007). Yet such sex differences are not universal: numerous taxa including multiple lemur species, bonobos ( *Pan paniscus* ), spotted hyenas ( *Crocuta crocuta* ), meerkats ( *Suricata suricatta* ), and elephants ( *Loxodonta africana* ) can exhibit greater levels of female aggression, co‐dominance between males and females, or even female‐biased dominance (Clutton‐Brock et al. 2006; Richard 1987; Surbeck and Hohmann 2013; Watts and Holekamp 2007; Wittemyer and Getz 2007). This indicates that the power dynamics between females and males can vary significantly across species depending on socioecological context (Clutton‐Brock and Huchard 2013b; Tombak et al. 2024). As such, cross‐species and intraspecific comparisons can provide insights into why sex differences in behavior emerge, as well as how malleable they are.

Among primates, wild chimpanzees ( *Pan troglodytes* ) show especially robust sex differences in social behavior (Goodall 1986; Lehmann and Boesch 2008; Muller and Mitani 2005; Wrangham and Smutts 1980). For example, rates of aggression among adult males are consistently higher than in adult females, and males specifically utilize aggression when competing among each other for status and for access to estrous females (Feldblum et al. 2014; Goodall 1986; Muller 2002). When female chimpanzees are aggressive, in contrast, they are mostly aggressive in the context of competition over food access or in response to immigrant females (Miller et al. 2014; Pusey and Schroepfer‐Walker 2013). These sex differences also extend into dominance and intersexual interactions: all adult males outrank all adult females in wild chimpanzee communities, likely in part because males are moderately larger than females (Leigh and Shea 1995; Pusey et al. 2005; Uehara and Nishida 1987). Finally, males and females use different processes to acquire rank: males rise in rank through overt aggressive competition, whereas females conversely ‘queue’ for rank (Foerster et al. 2016; Watts 2018).

Another striking feature of sex differences in chimpanzee behavior concerns patterns of social relationships. Unlike many primates who are female‐bonded, male chimpanzees are more gregarious than female chimpanzees both in terms of proximity to others and grooming patterns (Goodall 1986; Lehmann and Boesch 2008; Muller and Mitani 2005; Nishida 1968; Wittiger and Boesch 2013). In contrast, females spend far less time in proximity to other adults compared to males, and male–male grooming occurs more frequently than female–male or female–female grooming (Arnold and Whiten 2003; Grueter et al. 2023; Machanda et al. 2013; Surbeck et al. 2017). Males also exhibit a suite of cooperative behaviors with other males, including group hunting, territorial boundary patrols, and participation in coalitionary aggression in order to outcompete others or for mating access (Mitani et al. 2000; Muller and Mitani 2005; Nishida 1983; val Lawick‐Goodall 1968; Watts 1998). Females, conversely, more rarely engage in these joint behaviors (Gilby et al. 2017; Watts and Mitani 2001). For example, males frequently rely on coalition aggression, whereas coalitions are rare between female chimpanzees (Fox et al. 2022; Muller and Mitani 2005; Newton‐Fisher 2006).

What drives these stark sex differences in chimpanzee aggression and affiliation? A clear contributor is their mating system and pattern of offspring care, as their promiscuous mating exacerbates the intensity of male–male competition, and females engage in extended investment in offspring with long inter‐birth intervals (Emery Thompson 2013; Muller et al. 2007; Tutin 1979). In addition, the unusual system of male philopatry among chimpanzees favors stronger male–male social bonds, cooperation, and coalitions (Bray and Gilby 2020; Inoue et al. 2008; Langergraber et al. 2009; Mitani 2009). Importantly, one prominent idea is that these sex differences also stem in part from ecological constraints on female behavior. In particular, the impact of scramble competition for limited food resources is proposed to be especially costly for females who bear the energetic costs of caring for dependent infants, and these costs may preclude them from foraging together and thus forming social relationships with other adults (Wrangham 2000). Accordingly, female chimpanzees at East African sites are often characterized as being relatively asocial compared to males: they often range alone with just their dependent offspring (Gilby and Wrangham 2008; Wrangham and Smutts 1980) and overall are less socially integrated than males (Thompson Gonzalez et al. 2021).

In line with this proposal that sex differences in chimpanzee social behaviors arise in part due to constraints on females, intraspecific comparisons across chimpanzee communities highlight how ecological context may impact patterns of female sociality. For example, chimpanzees from the Taï community in Ivory Coast more often range with other adult males and females, and are generally thought to be more social than their East African counterparts (Boesch and Boesch‐Achermann 2000; Lehmann and Boesch 2008, 2009). This has been proposed to stem, in part, from the greater food availability in West African compared to East African sites (Doran 1997; Lehmann and Boesch 2005; Wittiger and Boesch 2013). Similarly, there is some evidence from some wild chimpanzee populations that females can be somewhat more gregarious when food resources are generally more available (e.g., reducing scramble competition), or when food is relatively clumped, and thus the energetic costs of socializing are reduced (Grueter et al. 2023; Wakefield 2008; Wittiger and Boesch 2013). Comparisons of party composition using camera traps further show that Western chimpanzees in Guinea had more female‐biased social parties, potentially because they were accessing high value crab‐fishing sites, compared to Eastern chimpanzees in Uganda (Koops et al. 2024). Finally, this socioecological hypothesis can also potentially explain why female bonobos exhibit strong social bonds, more frequent coalitions, and higher status despite also exhibiting a social organization involving promiscuous mating and male philopatry like chimpanzees (Wrangham 1986): in this proposal, female bonobos experience reduced constraints related to acquiring food compared to female chimpanzees (Malenky and Wrangham 1994; White 1989, 1998; White and Wrangham 1988), allowing them to form stronger social bonds, use coalitionary aggression against males, and ultimately acquire higher dominance status (Furuichi 2011; Marchant et al. 2002; Surbeck et al. 2025, 2017; Surbeck and Hohmann 2013).

Evidence from captive or provisioned chimpanzee groups also provides some converging evidence for this socioecological proposal, as provisioning means that these groups do not face the same foraging costs as chimpanzees in the wild. In fact, observations of these groups indicate that females can engage in different patterns of socializing more comparable to males. For example, males and females in semi‐free‐ranging groups at Chimfunshi Wildlife Orphanage in Zambia showed similar grooming and association patterns across groups (Rawlings et al. 2023). Moreover, prior studies in zoos indicate that females in captivity may engage in more coalitionary aggression or acquire higher status than is typical in wild communities (Baker and Smuts 1994; de Waal 1984; Funkhouser et al. 2018; Spijkerman et al. 1997). In particular, observations of chimpanzees at the Arnhem Zoo in the Netherlands revealed that females will form coalitions, and that their patterns of support are based on their social bonds (de Waal 1984). However, some captive groups are relatively small or have very few adult males (e.g., the Arnhem study group had only 13 adults with a total of four adult males). As such, some of these patterns may arise in part due to demographic constraints rather than ecological context per se.

Overall, this evidence shows that chimpanzees provide an interesting test case for examining both the drivers and malleability of sex differences in social behavior. While male chimpanzees in the wild are always reported to be more aggressive than females, female chimpanzees' level of sociality appears to be more variable from one community to the other (Wakefield 2008). That is, although chimpanzees show relatively strong sex differences in behavior, some aspects of female sociality and status can shift depending on context. Chimpanzees are also marked by high levels of behavioral variation across populations (Kalan et al. 2020; Marchant et al. 2002), highlighting their general capacity for flexible social and ecological behavioral strategies across contexts. As such, intraspecific variation in chimpanzee social behavior can identify the factors shaping sex differences in behavior, and more generally inform an emerging picture of male–female power dynamics that is more nuanced (Davidian et al. 2022; Kappeler et al. 2022; Smith et al. 2020).

Here we leverage a naturalistic population of semi‐free‐ranging but provisioned chimpanzees to examine patterns of social behavior when ecological constraints are relaxed. Chimpanzees at Ngamba Island Chimpanzee Sanctuary, Uganda, live in a large group (~50 individuals) with numerous adult males and adult females, similar to many wild communities. They also range on a large, forested island (~95 acres of space) with chimpanzee‐appropriate habitat, which allows them to make social choices about their close associations in line with fission‐fusion patterns in the wild. However, unlike in the wild, these chimpanzees are fed such that all chimpanzees obtain an appropriate amount of food based on their individual body size and needs, and thus changes in the composition of the group do not impact individual food acquisition. This reduces or eliminates the forms of scramble competition for insufficient resources that are common in the wild and which are a key factor thought to impact female sociality. Moreover, females are on birth control and therefore have few infants and associated costs of infant care. As such, proposed socioecological constraints on females' ability to socialize due to the costs of foraging along with caring for infants should be relaxed in this context. Here, we used behavioral data on sanctuary chimpanzees (Rosati et al. 2023) collected with methods directly adapted from wild protocols used to observe the Kanyawara community at Kibale National Park, Uganda (see Emery Thompson et al. 2020 for details of this wild study site). We specifically compared male and female social behavior in this group to assess the direction and strength of sex differences, including (1) patterns of affiliation (proximity and grooming); (2) patterns of aggression (including displays, directed aggression, and coalition formation); and finally (3) both same‐sex and inter‐sexual dominance relationships. In line with the socioecological hypothesis for chimpanzee social relationships, we predicted that the increased food availability in this population would allow females to show relatively more affiliation, engage in more coalitionary aggression, and obtain higher social status relative to typical patterns in the wild. Conversely, we predicted that males would exhibit more aggressive behavior overall compared to females, as is the case in the wild, given that energetic constraints are not thought to strongly impact this aspect of male behavior.

## 2 Methods

### 2.1 Ethics

This study was conducted at Ngamba Island Chimpanzee Sanctuary, a Pan African Sanctuary Alliance accredited sanctuary in Uganda. Research was approved by the Uganda Wildlife Authority, the Uganda National Council for Science and Technology, and the Institutional Animal Care and Use Committee at the University of Michigan. Research practices and animal care procedures complied with guidelines from Ngamba Island Chimpanzee Sanctuary and Pan‐African Sanctuary Alliance standards.

### 2.2 Study Population

We examined the social behavior of 45 chimpanzees (17 males and 28 females; ranging from age 11–37 years) over a two‐year period at Ngamba. Chimpanzees included in this study were primarily adults as all individuals in the study, except one, were 15 years or older. 15 years is a standard cutoff for adulthood for males in the wild and corresponds with the typical age males are part of the adult dominance hierarchy (Enigk et al. 2021; Machanda et al. 2013). The only individual in the group between the ages of 10 and 15 during this observation period was a female who was 11–12 years during the two‐year observation period. We included her in the study as many wild studies consider females to be adults at sexual maturity, which can occur around this age (Walker et al. 2018), and this female routinely interacted with the adults (the other juveniles and infants in the group were several years younger). These 45 individuals lived in a single group that also included 5 younger infants and juveniles (for a total of 50 chimpanzees in the forest group during the study period; two additional adult males at the sanctuary interacted with the main group at night but were cared for in a separate group during the day and were not included in this study).

Most sanctuary individuals were wild‐born orphans who were integrated into conspecific social groups on arrival to the sanctuary around ages 1–3 years old. Prior work has shown that African sanctuary chimpanzee populations, including the Ngamba community, have healthy patterns of cognition, behavior, and physiology compared to many other captive contexts (Cole et al. 2024, 2020; Rosati et al. 2023; Wobber and Hare 2011). Importantly, adult female chimpanzees at the sanctuary are on hormonal birth control implants, so females have few infants. Six individuals in the group were born at the sanctuary due to birth control failures (1 adult included in the current study, as well as 2 juveniles and 3 infants whose behavior was not analyzed). Two adult females were absent from the primary group for a period of approximately 1 year in the middle of the study period during pregnancy and birth; observations for these individuals were included from the periods they were present in the forest, as described below.

The study group semi‐free‐ranged in approximately 40 ha of species‐appropriate tropical forest every day, and voluntarily entered a dormitory to sleep and receive supplemental feedings at night. Chimpanzees in this population can forage for a variety of plant food in the tropical rainforest habitat based on seasonal availability, including fruits and/or leaves from fig trees (*Ficus ovata* and *Ficus natalensis* ); wild ginger (*Aframomum* spp.); forest fever trees (*Anthocleista grandiflora*); incense trees ( *Canarium schweinfurthii* ); and Christmas bush (*Alchornea cordifolia*). However, a large portion of their daily food is provisioned. Approximately four times daily, caretakers provide the chimpanzees with a variety of domesticated fruits and vegetables including bananas, pineapple, watermelon, oranges, papaya, carrots, sweet potato, cabbage, and various leafy greens; they also receive limited amounts of processed foods such as cornmeal or millet porridge. Three times per day while chimpanzees are in the forest, caretakers provide this food by tossing food items into the enclosure over the fenceline. During the provisioning, caretakers aim to ensure each chimpanzee get sufficient food by distributing an appropriate allotment to each individual (i.e., food is not provided as a clumped or monopolizable resource). In the evening, chimpanzees who choose to voluntarily enter the dormitory also receive provisioned foods (here, each chimpanzee is directly handed food by caretakers standing outside the dormitory, again to ensure each individual gets their specific allotment). Some chimpanzees chose not to enter the building at night, and rather sleep in the forest (presumably eating forest foods at night). To ensure the wellbeing of the chimpanzees, caretakers confirm that they have seen each chimpanzee daily and that they are in good health, regardless of whether they enter the building at night (e.g., chimpanzees who sleep outside are typically still seen with the group during the daytime).

### 2.3 Observational Methods

We conducted two years of observations comprising 10‐min focal follows modeled after data collection methods and behavioral definitions from the Kibale Chimpanzee Project (Emery Thompson et al. 2020). Focal follows were collected by trained caretakers at the sanctuary who received extended training on observational methods, including direct oversight by an experienced field assistant from the Kibale Chimpanzee Project (see Rosati et al. 2023 for a detailed description of the initiation of this larger project, including adaptation of observational methods from the wild). As observers cannot follow the sanctuary chimpanzees in their forest enclosure in the absence of a barrier due to safety reasons, as would be typical with wild populations, focal follows were collected from observation platforms and areas adjacent to the forest fence line where caretakers provision the chimpanzees and could routinely see chimpanzees in the forest (e.g., these areas are set up in part for tourists to observe the chimpanzees).

For each ten‐minute focal, we recorded information about the individual's activity state (e.g., resting, traveling, feeding, grooming, or playing) every two minutes along with the identity of any individuals within 1 m proximity of the focal individual on those scans. This 1 m proximity measure is our first measure of individual differences in association. We chose to examine 1 m proximity as a meaningful measure (e.g., rather than 5 m proximity, a more common measure in wild chimpanzees) as part of the larger study because the chimpanzees are often more densely distributed in this population than in the wild (see Rosati et al. 2023). We did not collect systematic party membership data in these scans. Wild chimpanzee sites typically define a chimpanzee party using a chain rule where no individual in the same party can be more than 50 m apart (Clark and Wrangham 1994; Mitani et al. 2022), whereas some work in other African sanctuaries has defined party membership as individuals within 10 m, reflecting their greater density (Rawlings et al. 2023). However, because the majority of this chimpanzee group is at the observation areas during the observation time points, and they all have visual and auditory contact with each other (albeit in spaces that could exceed 50 m) we thought this was not a meaningful index of association in this context (see also Rosati et al. 2023). As such, we considered the close proximity measure—effectively cases where the chimpanzees choose to be within arm's reach of each other, also paralleling metrics of close proximity in other studies in sanctuary‐living chimpanzees (Rawlings et al. 2023)—to best capture differentiated social relationships between chimpanzees in this population.

In addition, we recorded all occurrences of grooming and aggression. Instances where the focal individual engaged in grooming was our second measure of association. Here, we recorded the duration of the bout and details including partner identity, direction of grooming (giving or receiving), mutual grooming, and chain grooming (Machanda et al. 2013, 2014). Second, we recorded all occurrences where the focal individual was involved in aggression following definitions from the Kibale Chimpanzee Project (i.e., as in Muller 2002; Muller et al. 2021), including when they engaged in non‐directed aggression (e.g., displays) versus directed aggression (e.g., aggression with a victim); the type of aggression (e.g., threats involving lunges or chases, versus attacks involving contact aggression); whether the aggression involved a coalition (simultaneous aggression by two or more individuals directed at one or more victims); and finally the victim identity and victim response (e.g., if the victim submits such as by screaming and fleeing; if they retaliate by directing aggression back to the aggressor, or if they have neutral response). Third, we recorded all occurrences where the focal individual gave or received a pant grunt or pant bark (a submissive vocalization that is a formal signal of dominance in chimpanzees; chimpanzees typically give this vocalization when a higher ranked individual approaches them, as opposed to as a response to received aggression). Finally, if individuals went out of view during the ten‐minute period (e.g., entered the forest where observers could no longer see them) we also recorded the time and duration of being out of view to be able to calculate behavioral rates, as described below. Additional data were also collected during these scans (such as on patterns of object manipulation, tool use, and various welfare indices), but were not analyzed as part of the current project focused on sex differences in social behavior (see Rosati et al. 2023 for more details about these aspects of the data collection).

During ten‐minute focals, observers chose an individual chimpanzee who had not been the subject of a focal follow that day, using a check sheet which tracked focals over time to equalize observation effort for each chimpanzee in each timeslot. Specifically, observations were completed during four timeslots where the group was typically visible from the observation locations and caretakers had time to conduct observations: in the morning after the group was released from the night dormitory (approximately 8:00 AM), late‐morning (approximately 11:00 AM), afternoon (approximately 2:30 PM) and evening (approximately 5:30 PM). The first three timeslots correspond to periods when caretakers went to the observation platforms in order to provision the group; chimpanzees approached the observation area around these times to receive food that caretakers threw into the enclosure. In contrast, the final slot did not involve any provisioning and corresponded to a period before the group could enter the dormitory for the evening, when chimpanzees often congregated in the forest area and were visible. Observers could typically conduct ten‐minute focals on one or two individuals in each timeslot as feasible, before returning to their normal caretaking duties; successive focals were done on different individuals, and were guided by a check sheet provided approximately every 2 weeks to ensure that all individuals in the group were observed at similar rates. This check sheet identified high‐priority individuals who had not yet been observed in a particular timeslot in the most recent sets of scans, to ensure they were observed if possible. By selecting high‐priority individuals from the check sheet, we also reduced bias of having observers select individuals engaged in interesting behaviors as the next focal subject. We tracked observations of individuals at particular timeslots in addition to overall individual observation effort as we expected observation timeslot to impact the chimpanzees' behavioral patterns based on prior analyses, such as with more aggression during provisioning contexts earlier in the day (Rosati et al. 2023), as also discussed more below.

Finally, approximately once per day we conducted a reliability scan where a focal individual was observed by two independent observers simultaneously; this allowed us to calculate reliability measures for our observational data (see below for more details). The final dataset then included one of these observers' focal scans; observers had high reliability on the core aspects of the data (see below), so we typically incorporated the focal scan from the observer who had more detailed longhand comments about relevant events. As relevant, we then also incorporated details from both observers in a ‘merged’ scan that was incorporated into the final dataset (e.g., if one observer noted additional features such as the identity of additional individuals involved in a complicated social interaction with multiple participants).

### 2.4 Social Metrics

We calculated several metrics of individual behavioral rates and dyadic interactions between the 45 target chimpanzees (that is, excluding interactions with the five infants and juveniles also in the forest group). All metrics were calculated from focal data collapsed across the two years. We used this two‐year range as our initial checks indicated stable behavioral patterns across this period of data collection; prior work from the wild also has used two year periods for relevant comparisons and shown that doing so can provide sufficient data while also capturing relevant patterns of association (Gilby and Wrangham 2008; Machanda et al. 2013, 2014).

First, we used the two‐minute scans to calculate each focal's patterns of proximity with other adult individuals. We specifically looked at three metrics: individual proximity rate (number of scans where the focal was in proximity of at least one other chimpanzee/total in‐view scans for the focal); individual average proximity (the average number of total individuals with which the focal was in proximity across the two‐minute scans where they were in‐view); and dyadic proximity rate (the number of scans where the focal individual was within one meter of each possible partner/total in‐view scans for the focal). This dyadic metric allowed us to examine an individual's proximity patterns with all other possible male versus female partners; this again used only focal data, such that the data used to calculate dyadic proximity for a given focal with a given partner were independent of the data used to calculate proximity when their roles were reversed. Note that this dyadic approach first counts the focal's association with each other possible individual in the group, and only afterwards collapses across partner sex; this approach is therefore not biased by the fact that there are more females than males in the group, which is a demographic sex distribution also common in many wild communities (Emery Thompson et al. 2020; Lehmann and Boesch 2008; Mitani 2006; Nishida et al. 2003; Sugiyama 2004).

Second, we used the two‐minute scans to calculate each individual's patterns of grooming. We focused on individual total grooming rate (number of scans where the focal was giving or receiving grooming/total in‐view scans for the focal) and dyadic total grooming rate (number of scans where the focal was grooming with each possible partner/total in‐view scans for the focal); as with proximity, this dyadic rate used focal data and therefore comprised independent data for a given pairing depending on who was the focal. We also calculated comparable metrics for the focal giving grooming versus receiving grooming as a check; in these directional metrics, mutual (simultaneous) grooming was considered both giving and receiving grooming.

Next, we calculated several metrics of aggression. We calculated individual display rate (number of bouts where the focal engaged in non‐directed aggression/total in‐view scans for the focal), individual directed aggression rate (number of bouts where the focal directed aggression at a victim/total in‐view scans for the focal), and dyadic directed aggression rate (number of bouts where the focal aggressed a given partner as victim/total in‐view scans for the focal). We also calculated comparable metrics for bouts of directed aggression involving threats (e.g., lunges or chases) versus attacks (e.g., contact aggression such as hitting, biting, or kicking) to examine more specific details about the nature of male versus female aggression. Here, each discrete instance of aggression was considered a bout (e.g., an instance with multiple back‐and‐forth interactions between chimpanzees as part of the same interaction was considered a single bout; this would count as one total bout of focal directed aggression), and we then converted these rates into aggression events per hour based on number of in‐view two‐minute scans (note that all instances of the focal's involvement in aggression were recorded regardless of whether they occurred on such scans). The dyadic rate then allowed us to examine the identity and sex of the victims of aggression, e.g., whether males were more likely to direct aggression to female victims, similar to the dyadic analyses of proximity and grooming. Finally, we examined each focal's rate of coalition participation (count of directed aggression bouts with coalitions/total in‐view scans) as well as their proportion coalitionary aggression (count of directed aggression with coalitions/count of total directed aggression bouts by the focal) to examine patterns of aggression involving cooperation between two or more parties. The proportional metric accounted for variation in overall aggression rates, as captured by the directed aggression count. While our primary coalition analyses involved instances where the focal participated in the coalition, we also qualitatively examined the sex composition of all coalitions observed in this period, regardless of whether the focal was involved in the coalition.

Finally, we calculated David's scores (David 1987; Gammell et al. 2003) to assess each individual's dominance status. Here, we compiled all observations of decided, dyadic aggressive interactions (e.g., dyadic interactions where one individual clearly won the interaction because the other individual submitted, such as by running away and screaming, as opposed to retaliating or having a neutral response). We also compiled all instances of pant grunts or barks (a formal signal of subordinance often given without aggression occurring), following methods from the wild (Emery Thompson et al. 2020); here, the individual who received the pant grunt was considered the winner, as in analyses of wild dominance. Our primary analyses used both instances of aggressive outcomes and pant grunts as at Kibale (Muller 2002; Muller et al. 2021), but we also examined dominance patterns based on direction of pant grunts alone as an additional check. We then categorized individuals as high, medium, or low status for analyses based on these scores.

### 2.5 Statistical Analyses

We analyzed the chimpanzee's behaviors using linear mixed models in R version 4.5.2 (R Development Core Team 2025), implemented using the *lmer* function of the lme4 package (Bates 2010). For models of individual rates (e.g., of individual proximity, grooming, and aggression rates), models accounted for the focal individual's identity (as a random effect to account for repeated measures), the focal's age (continuous in years; here we used the individual's age at the midpoint of the data range for all analyses, since they collapsed over two years), and the data collection timeslot (as a factor), to account for the fact that in these data individuals tend to show different overall pattens of affiliation in different timeslots, as also reported in prior work (Rosati et al. 2023). In successive models we then added the focal's sex (male or female) to test our hypotheses about sex differences in this population, as well as the interaction between sex × timeslot to check if any observed sex differences were consistent across provisioning versus non‐provisioning contexts. We compared model fit using likelihood ratio tests (Bolker et al. 2008); these were implemented with the package *lmtest* for linear models. Post hoc comparisons (e.g., comparisons of factor levels) were conducted using the *emmeans* package (Lenth 2018) and implemented with Tukey corrections, and plots with model estimates were created using the *effects* package (Fox et al. 2016). We also checked model assumptions using the *DHARMa* package (Hartig 2022). Several of the models including timeslot as a predictor violated model assumptions about data distribution, likely because of the strong separation of data over the day (i.e., more affiliation occurred in the evening, whereas aggression was largely absent in the evening). We therefore checked these results using total rates over all observation time, using linear regressions that collapsed across timeslot (e.g., total count divided by total in‐view scans). Collapsing across timeslot addressed this distribution issue, and both of these approaches generated similar statistical outcomes in terms of sex differences in behavior. Analyses of coalition rates also collapsed across timeslots as these were relatively rare events.

Our analyses of dyadic interactions (e.g., between the focal and each other possible individual in the group) used a similar approach, here also testing how partner's sex impacted behavioral interactions. Specifically, these analyses allowed us to test if males and females might have a bias not just in how often they socialized, but also the sex of the other individuals with whom they socialized. Here, the base model accounted for the focal's identity and the partner's identity (both as random effects), the focal's age, and the age difference between the focal and partner to capture if the partner was relatively younger versus older than the focal. We then added the focal's sex, the partner's sex, and their interaction in successive models to test our hypotheses, and similarly compared model fit using likelihood ratio tests. As these models already split data across partner identities, we did not additionally account for timeslot here.

Finally, David's scores were calculated with the R package *EloRating* (Neumann and Kulik 2024). We used dyadic aggressive interactions with decided outcomes (e.g., one individual submitted to the other), as well as instances of pant grunts and pant barks (a formal signal of subordinance that commonly occurs without the occurrence of aggression), in these analyses. We first calculated the male and female dominance hierarchies separately using same‐sex interactions (e.g., males interacting with other males) as is standard in studies of wild populations (e.g., Foerster et al. 2016). We then calculated a full mixed‐sex hierarchy using all adult interactions in order to assess the relative positions of males and females and test whether all males outranked all females, versus whether the hierarchy was integrated across the sexes. Our primary approach was to use David's scores calculated from the dyadic proportion of all wins (P_ij_) to assign categorical ranks (high, medium, and low), as we had well‐equalized observation effort across individuals. We also checked analyses using (1) the dyadic proportion of wins corrected for chance (i.e., *D*_ij_) (de Vries et al. 2006), and (2) calculating *P*_ij_ based on only the records of pant grunts and pant barks (e.g., not including the outcomes of aggressive interactions). Finally, we examined the linearity (de Vries 1995) and steepness (de Vries et al. 2006) of these hierarchies using the *h*.index and steepness functions, respectively.

## 3 Results

### 3.1 Observation Effort and Reliability

Overall, the dataset consisted of a total of 6618 ten‐minute focal follows, comprising a total of ~1103 h of observation over the two years. A total of 6.7% of the ten‐minute focals in this dataset were reliability scans, and we found high reliability across all relevant measures (see Supporting Information for all details of the reliability checks). Chimpanzees were observed for an average of 813.3 two‐minute scans where they were in‐view. Across the sample, females were observed an average of 803.0 two‐minute scans (averaging 26.8 h of observation per female; range: 303–895 scans per individual female; two females were observed for only 303 and 353 scans respectively, as they were out of the main group for about half the observation period due to pregnancies as noted above, whereas all other females had at least 780 scans). Males had an average of 830.2 scans (averaging 27.7 h of observation per male; range 739–878 scans per male).

We examined observation effort using linear mixed models where the base model accounted for the focal's identity (as a random factor) and their age in years. Further inclusion of timeslot indicated that this improved model fit [*χ* ^2^ = 280.39, df = 3, *p* < 0.0001]: chimpanzees were overall observed most often in the evening (there was an average of 251.3 in‐view scans per chimpanzee in the evening slot), and least often in the morning (when individuals often quickly entered the forest and left visible areas where observers could conduct scans; there was an average of 115.1 in‐view scans per chimpanzee in the morning slot); they were observed at similar intermediate rates in the late‐morning and afternoon timeslot (an average of 224.9 and 222.0 scans per chimpanzee, respectively; *p* < 0.0001 for significant comparisons in post hoc tests). However, there was no additional model improvement by including chimpanzees' sex [*χ* ^2^ = 0.625, df = 1, *p* = 0.43, n.s.; see Supporting Information for model parameters]. The further inclusion of an interaction between sex × timeslot also did not significantly improve model fit compared to the model with only timeslot [*χ* ^2^ = 8.47, df = 4, *p* = 0.08, n.s.]; posthoc tests on this model indicated no difference in rate of observation for males versus females in any timeslot (*p* > 0.10 for sex comparisons in each individual timeslot). That is, there was no sex difference in the rate of observation by sex at any specific timepoint in the day, indicating that we successfully implemented similar observation effort across both males and females. As these models produced singular fit (indicating low variance between individuals), we also confirmed this result with comparisons of overall in‐view scans and similarly found no improvement by including sex [*χ* ^2^ = 0.62, df = 1, *p* = 0.43, n.s.].

### 3.2 Proximity

**FIGURE 1:**
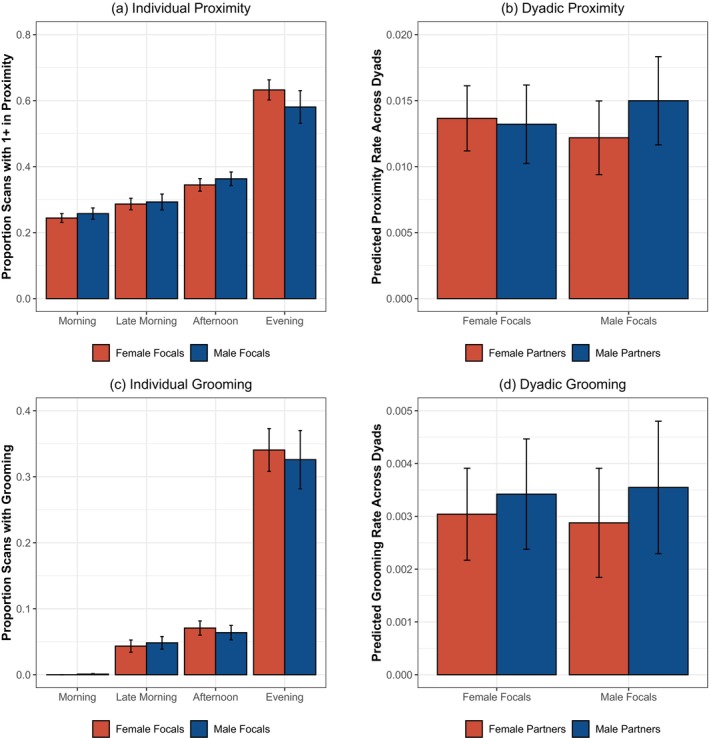
Patterns of affiliation in sanctuary chimpanzees. (a) Average proportion of scans where the focal is in close proximity to at least one other adult, split by observation timeslot and focal's sex; error bars indicate standard error. (b) Estimated effects of focal's sex and partner's sex on dyadic proximity rates, derived from mixed models also accounting for focal's age, the partner's age difference, and both individuals' identities; error bars indicate 95% CI. (c) Average proportion of scans where the focal was grooming (either giving or receiving), split by timeslot and focal's sex; error bars indicate standard error. (d) Estimated effects of focal's sex and partner's sex on dyadic grooming rates, derived from mixed models also accounting for focal's age, the partner's age difference, and both individuals' identities; error bars indicate 95% CI.

We next examined rates of close proximity with other adults, assessing whether male chimpanzees showed higher rates of association with others than females, as is the case in the wild (Machanda et al. 2013). Overall, females were observed within 1 m proximity to at least one other adult on mean = 40.6 ± SE = 1.7% of in‐view scans (range: 23.1%–58.4% of scans across different females), and males on an average of 39.7% ± 2.7% of scans (range: 15.6%–57.7% of scans across different males). We examined rates of proximity using linear mixed models where the base model accounted for the focal's identity (as a random factor), their age in years, and the observation timeslot; only timeslot was a significant predictor in the base model, and post hoc tests revealed rates of proximity increased over the course of the day with higher overall rates in the afternoon and evening timeslots compared to the morning and late‐morning timeslots, as well as more association in the evening compared to the afternoon (*p* < 0.01 for significant comparisons). Neither the additional inclusion of focal's sex [*χ* ^2^ = 0.005, df = 1, *p* = 0.94, n.s.; see Supporting Information for parameters from this model] nor the interaction between sex × timeslot [*χ* ^2^ = 4.22, df = 4, *p* = 0.38, n.s] further improved model fit compared to the base model. This indicates that while overall rates of proximity changed in the group over the day, males and females showed similar rates of proximity at each observation time (see Figure 1a).

As checks of this result, we implemented several additional models examining: (1) overall proximity rates collapsing across timeslot; (2) the average total number of other adults that focals were in proximity at each two‐minute scan; and (3) the proportion of 2‐min scans at which individuals were observed ‘alone’ with no one in close proximity. Across all of these analyses, we similarly found that males and females did not show significantly different patterns (see Supporting Information for all details of these analyses). For example, in the evening scans where individuals generally showed the highest rates of close proximity, females were observed in proximity to an average of 1.04 total other adults (range: 0.48–1.70 individuals on average across different female focals), and males with 0.96 other adults (range: 0.28–2.01 on average across male focals). Overall, these analyses indicate that males and females showed very similar tendencies to be in close social proximity.

We further examined rates of dyadic proximity to test if males and females showed sex‐based partner preferences, as wild male chimpanzees not only show higher proximity rates than females but also specifically spend time near other males at higher rates (Machanda et al. 2013). In this analysis, we examined the rates of the focal being in proximity to each other possible individual in the group, and then tested if focal's sex and/or their partner's sex impacted this likelihood. Across these models, inclusion of the focal's sex did not improve fit compared to a base model including focal's identity and partner's identity (both as a random effects), focal's age, and the age difference between the focal and the partner [*χ* ^2^ = 0.05, df = 1, *p* = 0.83, n.s.]; the only significant predictor in this analysis was focal's age, with older individuals showing higher proximity rates to other individuals than did younger individuals. Additional inclusion of an interaction between focal's sex × partner's sex also did not improve fit compared to the base model [*χ* ^2^ = 6.23, df = 3, *p* = 0.10, n.s.; see Supporting Information for parameters from this model], indicating no overall bias towards same versus same‐sex versus opposite‐sex partners for either males or females (see Figure 1b). Notably, the top three proximity dyads in the group were all female–female dyads, also aligning with the interpretation that females were quite sociable in this group. Overall, this again provides no evidence for a male bias in social affiliation: males and females showed similar rates of association overall and with both other males and females.

### 3.3 Grooming

We then examined rates of grooming, to assess if male chimpanzees show higher rates of grooming compared to females as in the wild (Lehmann and Boesch 2008; Machanda et al. 2013). We first examined individual total grooming rate (all instances of the focal grooming in any direction), using the same analysis approach as for proximity. Overall, females were observed grooming on 13.8% ± 1.4% of in‐view scans (range: 2.6%–31.2% of scans across female focals), and males on 13.4% ± 1.8% of scans (range: 0.8%–25.2% of scans across male focals). As with proximity, timeslot was a significant predictor in the base model also including focal's identity: paralleling the patterns for close proximity, grooming increased over the course of the day (*p* < 0.01 for significant cases). However, neither the additional inclusion of focal's sex [*χ* ^2^ = 0.03, df = 1, *p* = 0.87, n.s.; see Supporting Information for parameters from this model] nor the interaction between sex × timeslot further improved model fit [*χ* ^2^ = 0.36, df = 4, *p* = 0.99, n.s.], indicating similar rates of grooming across males and females for all timeslots (see Figure 1c). Additional analyses examining (1) overall grooming rates collapsing across timeslot and (2) the specific direction of grooming (i.e., giving versus receiving grooming) found the same pattern: there were no sex differences in grooming (see Supporting Information for details). The only additional result was that older individuals were more likely to receive grooming than younger ones [estimate = 0.002, *t* = 2.76, *p* < 0.01], with no age effects in the other grooming metrics. Overall, this indicates that males and females showed similar tendencies to engage in grooming with others.

We then examined rates of dyadic grooming to assess with whom males and females groomed, again following the same logic and procedure as for the analyses of proximity. In models, inclusion of the focal's sex did not improve fit compared to a base model [*χ* ^2^ = 0.01, df = 1, *p* = 0.91, n.s.]; the only significant predictor here was focal's age, with older individuals showing higher grooming rates. Additional inclusion of the interaction between focal's sex × partner's sex also did not improve fit [*χ* ^2^ = 1.02, df = 3, *p* = 0.80, n.s.; see Supporting Information for parameters from this model], indicating that males and females were similarly likely to groom same‐ versus opposite‐sex partners (see Figure 1d). Notably, the top three grooming dyads in the group were also female–female dyads (in fact, these were the same individuals with the highest proximity scores, suggesting they had especially strong bonds). Additional analysis of dyadic grooming broken down by giving versus receiving grooming showed overall similar patterns of results, with no differences by sex; the only additional result was that the dyad's age difference was a significant predictor specifically for giving grooming (e.g., older focals both gave more grooming, and preferentially groomed individuals who were relatively older than they were; see Supporting Information). Overall, this provides no evidence for a male bias in grooming: as was the case for proximity, males and females show similar rates of grooming overall, and some female dyads showed quite high rates of grooming.

### 3.4 Aggression

**FIGURE 2:**
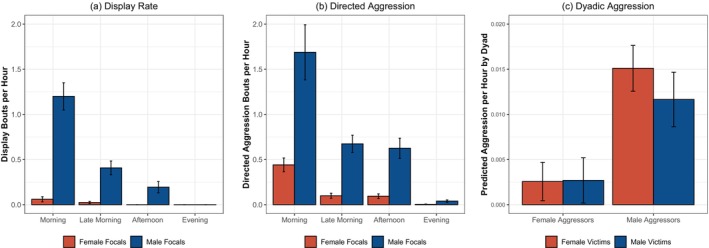
Patterns of aggression in sanctuary chimpanzees. (a) Average rate of focal's displays per hour, split by observation timeslot and focal's sex; error bars indicate standard error. (b) Average rate of focal's directed aggression per hour, split by observation timeslot and focal's sex; error bars indicate standard error. (c) Estimated effects of aggressor's sex and victim's sex on aggression rates, derived from mixed models also accounting for focal's age, the partner's age difference, and both individuals' identities; error bars indicate 95% CI.

We next examined patterns of aggression, to asses if males show higher rates of aggression than do females as in the wild (Muller 2002). We specifically examined the focal's engagement in non‐directed aggression (displays) and directed aggression (e.g., threats, chases, and attacks directed at others), using the same basic analysis approach as for proximity and grooming. Overall, female focals engaged in an average of 0.015 ± 0.006 displays per hour of observation (range: 0–0.14 events per hour across female focals), whereas males engaged in an average 0.33 ± 0.047 displays per hour (range: 0.068–0.67 events per hour across male focals). While all males were observed displaying at least once, 21 of the females were never observed displaying. Timeslot was again a significant predictor in the base model: displays occurred most frequently in the morning timeslot relative to all other timeslots, and decreased over the course of the day to similarly low rates in the late morning, afternoon, and evening (*p* < 0.0001 for significant cases). Here, the additional inclusion of focal's sex did improve model fit [*χ* ^2^ = 50.73, df = 1, *p* < 0.0001; see Supporting Information for parameters from this model]: males overall displayed more than females. Finally, the inclusion of the interaction between focal's sex × timeslot also improved fit compared to the second model [*χ* ^2^ = 118.2, df = 3, *p* < 0.0001]; posthoc tests showed that males displayed more than females specifically in the first three timeslots (*p* < 0.01 in all cases), but that males and females showed similar low display rates in the evening timeslot (see Figure 2a). We also conducted additional analyses examining overall display rates collapsing across timeslot, and found similar results with males displaying more than females overall (see Supporting Information). Overall, this accords with data from the wild showing that males engage in more display and overall dominance striving than females (Foerster et al. 2016).

We saw a similar pattern for directed aggression (bouts involving threats and/or attacks directed at victims): females engaged in an average of 0.11 ± 0.017 aggressive bouts with a victim per hour of observation (range: 0–0.36 events per hour across female focals), whereas males engaged in an average of 0.60 ± 0.069 aggressive events directed at a victim per hour (range: 0.14–1.0 events per hour across male focals); all male were again observed engaging in directed aggression, whereas here all but three females engaged in directed aggression at least once. Timeslot was again a strong predictor in the base model: directed aggression was highest in the morning timeslot, and also higher in the late morning and afternoon compared to the evening [*p* < 0.05 for significant cases]. The inclusion of focal's sex improved model fit compared to this base model [*χ* ^2^ = 38.19, df = 1, *p* < 0.0001; see Supporting Information for parameters from this model]: males engaged in more directed aggression than females. Finally, the additional inclusion of the interaction between focal's sex and timeslot also improved model fit compared to the second model [*χ* ^2^ = 36.25, df = 3, *p* < 0.0001]; post hoc tests indicated that, as with displays, males showed more directed aggression than females in the first three timeslots (*p* < 0.005 in all cases), whereas males and females showed similar low rates of aggression in the evening timeslot (see Figure 2b). We also conducted additional analyses examining (1) overall aggression rates collapsing across timeslot and (2) rates of threats versus attacks (e.g., distinguishing between directed aggression involving lunges or chases versus physical contact), and found the same patterns: males consistently showed more aggression than females (see Supporting Information). Overall, this indicates that this group exhibited a male bias in aggression, mirroring wild patterns.

We then examined rates of dyadic directed aggression, again following the same basic dyadic analysis approach as for proximity and grooming but here focused on directed aggression with a victim. Inclusion of the aggressor's sex improved model fit compared to a base model accounting for aggressor's and victim's identities (as random effects), aggressor's age, and the age difference between the aggressor and victim [*χ* ^2^ = 44.82, df = 1, *p* < 0.0001]: males aggressed more than females. Additional inclusion of the interaction between focal's sex × victim's sex trended to further improve fit [*χ* ^2^ = 5.75, df = 2, *p* = 0.056; see Supporting Information for parameters from this model]. Post hoc tests on this interaction indicated that (1) male focals aggressed both male and female victims more often overall than female focals did (*p* < 0.001 for both cases), (2) male focals aggressed female victims more than male victims (*p* < 0.05), but (3) female focals aggressed male and female victims at similar rates (*p* = 0.93, n.s.). Overall, this indicates that while the males were overall more aggressive than females and also targeted female victims more than males as in the wild, females aggressed both other females and males at similar rates when they did engage in directed aggression (see Figure 2c). Additional checks showed that this pattern of male aggressors targeting female victims more often than male victims was specifically driven by rate of threats as opposed to attacks (see Supporting Information).

### 3.5 Coalitions

**FIGURE 3:**
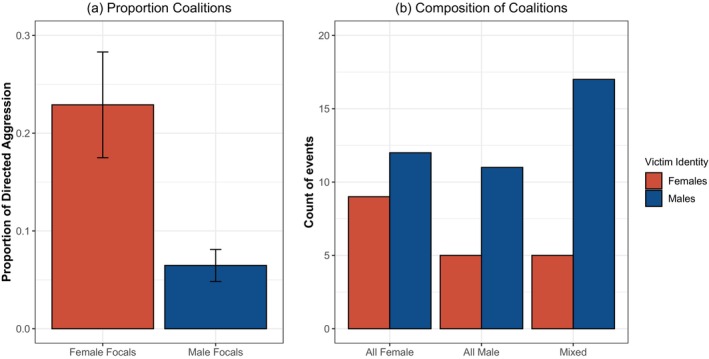
Coalitions in sanctuary chimpanzees. (a) Average proportion of directed aggression involving coalitions, split by focal's sex; error bars indicate standard error. (b) Overview of all coalitions in the observational dataset (e.g., regardless of whether the focal was in the coalition), split by composition of the coalitionary aggressors and the sex of victims; male victim sex here indicates at least one victim was male (one coalition aggressed both a male and female victim together, which was here grouped with the male victims).

We then examined patterns of coalitionary aggression, as male chimpanzees engage in more coalitions than do females in the wild (Fox et al. 2022; Gilby and Wrangham 2008). Overall, females engaged in coalitions at an average rate of 0.02 *±* 0.005 events per hour (range: 0–0.10 across female focals), and males engaged in coalitions at a rate of 0.04 *±* 0.008 events per hour (range: 0*–*0.11 across male focals). Overall, a total of 25 individuals were observed to participate in at least one coalition when they were a focal individual (14 of the 28 females, and 11 of the 17 males). As coalitions were rare events, we used linear regression models that collapsed across timeslots. Here, inclusion of aggressor's sex did not improve model fit, indicating similar overall rates of participation in coalitions for males and females as a function of observation time [*χ* ^2^ = 1.81, df = 1, *p* = 0.18, n.s.; see Supporting Information for parameters from this model]. Notably, males also had higher rates of directed aggression as noted above, suggesting that females' aggression actually involved a higher proportion of coalitions than did males. Indeed, when considering only individuals who ever engaged in focal aggression, an average of 22.9% of females' directed aggression bouts involved coalitions, but only 6.5% did for males. In line with this, models of proportion of coalitionary aggression (e.g., dividing bouts of coalitions by total bouts of directed aggression) showed improved fit when aggressor's sex was included in models [*χ* ^2^ = 5.78, df = 1, *p* < 0.05; see Supporting Information for parameters from this model], with a higher proportion of directed aggression involving coalitions for females compared to males (see Figure 3a).

We also qualitatively examined the composition of all observed coalitions regardless of whether the coalition included the focal (e.g., in some of these cases the focal was the victim in the interaction). Since our project focuses on focal aggression, these results should be taken with caution, but can also provide context for female involvement in coalitions. Overall, we recorded a total of 59 coalitions between adults over the two years: 16 were all‐male coalitions, 21 were all‐female, and 22 involved aggressors of both sexes (see Figure 3b). This accords with the focal rate analyses in showing that female actors were frequently involved in coalitions; here there was at least one female involved in 73% of all instances of adult coalitionary aggression observed in the group. There were also an additional three coalitions observed that involved a mother‐reared juvenile aggressing adult female victims with adult coalition partners (one of these instances involved the mother as the coalition partner, whereas the other two involved different adults); we do not further consider these instances here.

Among the coalitions involving only female adult aggressors, 12 (57%) aggressed a male; these were mostly the lowest‐ranking adult males (see below for details on the dominance hierarchy). In contrast, more of the mixed‐sex and all‐male coalitions aggressed males, and several of these male victims were higher‐status in the dominance hierarchy (69% of male‐led coalitions involved a male victim, and 77% of mixed sex coalitions did). All of the male‐led coalitions involved only two aggressors, whereas coalitions involving females sometimes involved up to four identified aggressors (three of the all‐female and mixed coalitions also involved additional group members of unknown identity, and in all three of these cases the group attacked a male victim). In considering the context of these coalitions, 45 (76%) of them involved a situation where the coalition protected or supported the victim of a prior aggression (in most cases that victim was one of the coalition partners and retaliated against their original aggressor). This included 9 of the male‐led coalitions (56%), 16 of female‐led coalitions (76%), and 20 of mixed sex coalitions (90%). Overall, this suggests that a high proportion of coalitions involving female aggressors overall targeted males and occurred in the context of protecting a prior victim.

### 3.6 Dominance

We next analyzed patterns of dominance in the group. As noted previously, to do so we compiled all instances of pant grunts or pant barks, as well as decided dyadic aggressive interactions with a clear winner and loser such that one individual clearly submitted to the other. We started by examining the male and female hierarchies separately using only same‐sex interactions. Between males, we observed 123 pant grunts and 125 decided aggressive outcomes, whereas between females we observed 25 pant grunts and 43 decided aggressive interactions. This pattern—of more frequent dominance interactions between males and fewer between females—aligns with patterns from the wild (Foerster et al. 2016; Wakefield 2008).

**FIGURE 4:**
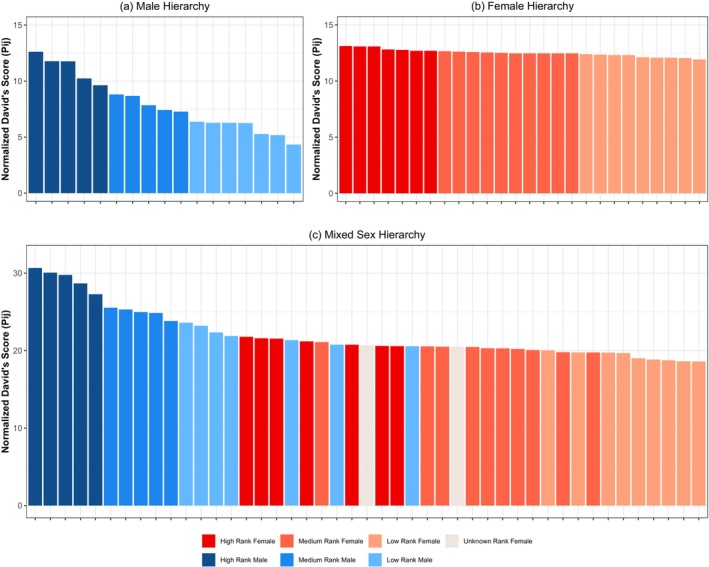
Dominance in sanctuary chimpanzees. The (a) male‐only and (b) female‐only same‐sex hierarchies, calculated from direction of decided aggression and pant grunts between same‐sex individuals. Individuals are then split into high, medium, and low categorical ranks based on these David's scores. Two females did not have any same‐sex interactions to include in the female‐only hierarchy. (c) The mixed‐sex hierarchy calculated from direction of decided aggression and pant grunts between all adults; each individual's color coding is derived from their categorical rank in their same‐sex hierarchy. Two females with unknown ranks in the female‐only hierarchy could be assessed in the mixed‐sex hierarchy, as they had interactions with males.

Our primary analyses involved generating David's scores using *P*_ij_, given that we had very equivalent observation effort across individuals. We first assessed the male and female same‐sex hierarchies and split chimpanzees into high, medium, and low rank based on these within‐sex scores (see Figure 4a,b for the same‐sex hierarchies). We also confirmed that the assignment of individuals to high, medium, and low rank categories derived from *P*_ij_ (involving raw proportions) were the same as those from *D*_ij_ scores (adjusting for chance), which was true for both sexes. For males, the hierarchy derived from pant grunts alone was also largely similar (one medium‐ranked male moved up to high rank and two low‐ranked males moved to medium rank when only pant grunts were considered). For the female hierarchy, two females—specifically the two who had been out of the group for a period due to pregnancies—had insufficient within‐sex interactions to generate David's scores for any of the female hierarchy analyses. When only pant grunts were considered for females, an additional 10 females could not be included given the low number of female‐to‐female pant grunts. Nonetheless, for the 16 females that could be included in this additional check focused on female pant grunts, the pattern was also largely similar to those results generated from analyses also including decided aggressions (there was only one category change where one low‐ranked female moved to medium rank in this assessment).

For both the male and female hierarchies, we also examined the linearity (Appleby 1983; de Vries 1995) and steepness (de Vries et al. 2006) of the calculated David's scores. These checks showed that the male hierarchy exhibited greater linearity and steepness than expected based on chance (linearity *h* index = 0.64, *h*′ = 0.69, expected *h* = 0.16, *p* = 0.001; steepness = 0.33, expected steepness = 0.13, *p* = 0.001). In contrast, the female hierarchy was somewhat linear but fairly flat (linearity *h* index = 0.08, *h*′ = 0.17, expected *h* = 0.11, *p* = 0.10, n.s.; steepness = 0.022, expected 0.019, *p* = 0.11, n.s.).

Finally, because we were interested in the relative status of males versus females, we then examined the mixed sex hierarchy, comprising all possible interactions (both the same‐ and mixed‐sex interactions). Mixed‐sex interactions comprised the majority of the observed dominance interactions (852 total). Breaking this down by type, we observed no instances where a male gave a pant grunt to a female, but 467 instances where a female directed this vocalization to a male. Conversely, while males won against females in 380 aggressive interactions, we observed 14 interactions where a female won in aggression against an adult male in a dyadic aggressive interaction; 10 of these interactions involved the three lowest‐ranking adult males identified in the male hierarchy. Calculation of David's scores for the mixed‐sex hierarchy using *P*_ij_ showed that nine females were higher‐ranking than at least one of those lowest‐ranking males (see Figure 4c). Examinations of this mixed‐sex hierarchy showed linearity patterns intermediate between the male‐only and female‐only hierarchies (linearity *h* index = 0.39, *h*′ = 0.43, expected *h* = 0.07, *p* = 0.001), and steepness also intermediate between the male and female hierarchy (steepness = 0.14, expected steepness = 0.05, *p* = 0.001).

In the mixed sex hierarchy, there was more variability depending on what analytic approach we used. However, crucially all analyses indicated that at least some females outranked these lower‐status males. For example, David's scores using *D*_ij_ indicated that 11 females were higher‐ranking than at least one male, and the analysis of pant grunts alone indicated that 14 females were higher‐ranking than at least one male. This might seem surprising given that no male was actually observed giving a pant grunt to a female (as opposed to in the primary analyses including aggression outcomes, where females sometimes won in dyadic fights). An important point here is that dominance calculations are based on the dyadic patterns of individuals' interactions with all other individuals, and scores are weighted based on the opponent's strength. For example, the highest‐ranking female in the mixed sex hierarchy based on pant grunts was observed giving a total of 25 pant grunts, but all were directed to the three highest‐ranking males (indeed, 21 of them were directed just at the second‐ranked male). Conversely, one of the low‐ranking males in the pant grunt mixed hierarchy was observed to give a total of 23 pant grunts, a similar total number, but directed these vocalizations to a total of 7 other males—including individuals characterized as medium rank in the male‐only hierarchy. Indeed, the individual he vocalized to the most frequently (12 times) was a medium‐ranked male. Similarly, another high‐ranking female in the mixed pant grunt hierarchy was observed giving 15 pant grunts only to the 5 males categorized as high‐rank, whereas the lowest‐ranking male in this hierarchy was observed giving 14 pant grunts to a total of 7 other males, and again some of these males were medium‐rank.

## 4 Discussion

Wild chimpanzees are a species known for extremely sex‐typed behavior, with males showing more aggression as well as more affiliation in the wild. We found that these sex differences in affiliative behavior are reduced in provisioned chimpanzees living in a large, semi‐free‐ranging social group. Specifically, female chimpanzees in our study exhibited patterns of gregariousness that were statistically indistinguishable from males, showing major differences from patterns typically observed in the wild (Gilby and Wrangham 2008; Machanda et al. 2013). We further found that while males were consistently more aggressive than females both in terms of non‐directed and directed aggression, as is the case in the wild (Muller 2002; Muller and Mitani 2005; Sabbi et al. 2021; Wrangham and Smutts 1980), females in this semi‐free‐ranging population directed their aggression at similar rates towards both male and female victims, unlike patterns seen in the wild (Muller 2002). Additionally, a high proportion of the aggression that females engaged in involved coalitions, also unlike in the wild (Fox et al. 2022). This is in line with prior work suggesting that higher levels of female gregariousness may allow for female coalition formation (Newton‐Fisher 2006). Following this pattern of increased social support for females in this group, around ~30% of the adult females outranked at least one adult male in mixed‐sex dominance analyses. Overall, this shows that at least some characteristic sex differences in chimpanzee sociality are malleable—and may even be ameliorated (e.g., the lack of sex differences in grooming and proximity) in some socioecological contexts.

Our findings are consistent with the hypothesis that ecological constraints in the wild limit female chimpanzee sociality (Wrangham 2000). As this population is provisioned, scramble competition for insufficient food resources has been minimized, thus reducing the energetic costs of socializing for females and potentially allowing for greater investment in social relationships. In our study, chimpanzees of both sexes engaged in similar levels of close social proximity and grooming with other adults, and even showed similar socializing with both same‐ and opposite‐sex partners. This is the most direct prediction from the socioecological hypothesis, given that these chimpanzees are each given sufficient amounts of food. Our results showing similar levels of association between males and females align with prior work from other African sanctuaries where chimpanzees live in similar semi‐free‐ranging conditions, but using different observational approaches (Rawlings et al. 2023). This also converges with evidence from the wild that social behavior in female chimpanzees can show intraspecific variation due to ecological conditions—increased food availability, or more clumped food resources, can reduce the costs of scramble competition and allow individuals to feed in groups or more easily reunite after feeding, thereby allowing females in some wild populations to be more sociable (Boesch and Boesch‐Achermann 2000; Grueter et al. 2023; Lehmann and Boesch 2008, 2009; Wittiger and Boesch 2013). Also in line with this ecological explanation, sex differences in bonobos are quite different from chimpanzees even though both species exhibit male philopatry with female immigration and thus have similar social systems: female bonobos are extremely gregarious, engage in frequent coalitionary aggression, and exhibit co‐dominance with males (Wrangham 1986; Furuichi 2011; Gruber and Clay 2016; Marchant et al. 2002; Surbeck et al. 2025, 2017). In that sense, the female chimpanzees in this provisioned population also show some partial parallels with female bonobos in terms of their greater investment in social relationships, participation in coalitions, and somewhat higher social status.

Beyond association with other adults, females in this context were also able to participate in coalitions with both males and females, in some cases even participating in coalitions targeting high‐ranking males. In the wild, female chimpanzees rarely engage in coalitions, participating at a much lower rate than males, and in some communities they do so primarily with other females with whom they have differentiated but only weak relationships because strong social bonds between females are rare (Fox et al. 2022). Furthermore, adult males in the wild overwhelmingly form coalitions with other males, and when they form coalitions with females as juveniles and adolescents it is almost exclusively with their mother (Enigk et al. 2020)—whereas these adult coalitions were unlikely to have involved kin given that individuals are primarily wild‐born orphans. This increased coalitionary participation by females in the sanctuary, including coalitions with males, might stem from increased female sociality providing more opportunities for coalition formation. In the sanctuary context, females are able to spend more time with other adults of both sexes, suggesting that they may form stronger bonds and receive more support than do wild females. An important question for future research is therefore to examine the composition and context of these coalitions. For example, our qualitative assessment suggested that females might especially participate in coalitions that involve protections in response to others instigating aggression, which is congruent with some work indicating that wild females primarily form coalitions to retaliate against male aggressors (Newton‐Fisher 2006), as opposed to proactively initiating aggression. A more in‐depth analysis of the coalitions at the sanctuary would be important to address these questions about why the Ngamba females form coalitions and whether this parallels the wild.

Females' increased affiliation and coalition participation may ultimately allow them to obtain higher rank in this group. In the wild, male chimpanzees who form more coalitions have more social support and are more socially central in the group also tend to have higher rank (Thompson Gonzalez et al. 2021; Watts 2018). Similarly, female bonobos have stronger bonds, engage in more coalitions, and obtain higher status than do female chimpanzees (Surbeck et al. 2025). In our analyses, we found that the male dominance hierarchy was more linear and steeper than the female hierarchy as in the wild, but we also conducted a novel mixed‐sex dominance analysis which showed some surprising results: some females won aggressive interactions against adult males and even had higher social status than those males. This result held both in the analyses using outcomes of aggressive events and patterning of pant grunts (a formal signal of submission), as the females who obtained the highest relative status in the mixed hierarchy primarily directed their pant grunts to only high‐ranking males. Thus, a key question for future research is whether strong bonds in female sanctuary chimpanzees also predict more coalitionary support and ultimately higher rank acquisition.

While these results show that some chimpanzee sex differences are malleable across contexts, it is important to note that many aspects of aggression and dominance in this group were similar to what has been reported in the wild. Specifically, males engaged in more displays and more directed aggression than females, and to some degree targeted female victims at higher rates than male victims. Such aggressive interactions are likely strongly biased by patterns of sexual dimorphism in body size and relevant physiological mechanisms like testosterone (Davidian et al. 2022; Muller 2002; Smith et al. 2020). Specifically, male chimpanzees are physically larger and have greater muscle mass than females (Emery Thompson et al. 2012; Leigh and Shea 1995), and such physical traits are likely to be strongly conserved across environmental contexts. Sex‐specific patterns in dominance rank also generally paralleled what we see in the wild, which is also not surprising in that fighting power (stemming in part from body size and strength) is key to dominance. For example, we recorded more dominance interactions between male dyads than between female dyads and found that the male hierarchy was steeper than that of females. This is in line with findings from the wild that males are more constantly and actively engaged in status striving than females (Foerster et al. 2016). Finally, we found that some females outranked some males, but these were the three lowest‐ranking males in the male‐only hierarchy, and all three were also relatively young adults (17–19 years). While there are no records of any adult female outranking an adult male in the wild, adolescent male chimpanzees begin their entry into the adult male hierarchy by first dominating each adult female (Enigk et al. 2021). As there is some variation in the age at which males succeed at dominating the highest ranked female, it is possible that males take longer in this ecological context to dominate those females given greater female sociality and social support. Thus, a key question concerns long‐term patterns of dominance interactions in this group as these younger adult males enter prime adulthood.

Across analyses, we found a strong effect of observation timeslot on the chimpanzees' patterns of behavior. As also previously noted, our observations show higher rates of aggression in the first three and especially the first morning timeslot, whereas affiliative behaviors tend to increase over the course of the day and peak in the evening timeslot. Wild chimpanzees also show clear patterning in their social behavior related to food availability and patch size (Anderson et al. 2002; Basabose 2004; Newton‐Fisher et al. 2000; Wrangham 2000), and we suspect that these trends in the current study may partially stem from the particulars of the chimpanzees' feeding regime. Specifically, the first three timeslots correspond to time periods when the chimpanzees approach observation platforms while receiving provisioned foods, which may incite conflict. Conversely, the evening slot does not involve provisioning, and chimpanzees tend to relax prior to entry to the building. Yet it is important to consider that while these diurnal patterns of social behavior make it challenging to directly compare rates of behavior with the wild, they do allow us to contrast male versus female behavior in directly matched situations. Our similar rates of observation effort for both males and females at each timeslot also are relevant, given that in the wild males may be habituated more easily than females (Emery Thompson et al. 2020), and females' lower rates of socializing means that they are seen less often especially in large, mixed‐sex parties. In the current dataset, we could directly compare male and female behavior in each timeslot with similar sampling density.

One important consideration for our study is how provisioning might directly impact patterns of social behavior. We have argued that provisioning is a key contributor to the patterning of sex differences in sociality because it reduces competition for insufficient food resources, but there are other ways in which the provisioning potentially could impact the chimpanzees' behaviors. We conducted several of our observational timeslots overlapping the periods when the group is being provisioned largely for practical purposes, as these are times when animal caretakers are able to pause their other duties to watch the group and when chimpanzees are often visible at the observation platforms. However, it raises the possibility that females show higher rates of proximity and grooming because they are actively clustered around food resources during these times. However, we note that the feeding regime does not involve a clumped resource (food is rather distributed to each individual to ensure they get an appropriate allotment), and our data further show that proximity and grooming rates are overall much higher in the evening without provisioning (and show no sex biases in any time slot). This indicates that females associate at high rates even when social choices are not plausibly driven by the immediate presence of food. Conversely, aggression rates are higher during the three provisioning timeslots, but they are especially high in the first scan of the day—suggesting that aggression rates are also not a simple product of the presence of food. We suspect the high rates in the morning are in part because chimpanzees are especially excited after they have first left the dormitory. In any case, we found consistent evidence that males engaged in more displays and more directed aggression than females across the entire day. More generally, our observational approach clearly does not capture everything these animals do in the larger island space, but given that observers cannot routinely enter the forest it would be necessary to implement a different research approach (such as camera traps; Koops et al. 2024) to observe the chimpanzees in other locations. However, we stress that our data do allow for directly matched comparisons of individuals' behaviors in the same context and build on prior work from the wild using ten‐minute focals to capture important variation, including sex differences, in chimpanzee behavior (Gilby and Wrangham 2008; Machanda et al. 2013).

It is also important to consider how the unique sanctuary context may otherwise contribute to the observed pattern of female gregariousness beyond provisioning. For example, females in this group are on birth control implants, following standard PASA guidelines for African sanctuaries. It is possible that these implants impact their behavior, although we note that prior work has not shown major behavioral impacts in direct assessments (Bourry et al. 2004; Moresco et al. 2020, 2021). Moreover, females in this group show reduced estrous swellings due to the birth control implants, and in the wild the presence of swollen females actually tends to increase party size and consequent opportunities for socializing (Anderson et al. 2002; Hashimoto et al. 2001; Wakefield 2008). This suggests that any behavioral effects of birth control may in fact work against the high rates of female socializing we observed here. Conversely, swollen females in the wild experience aggression from males related to sexual coercion (Feldblum et al. 2014; Muller 2002; Muller et al. 2007), and therefore close proximity to males can involve the risk of aggression. Females in this group may therefore be less wary of maintaining close proximity to males than in the wild given their reduced swellings. However, our data show that females are in fact the preferential victims of male aggression in this population, so females still face a salient risk of aggression from males in this group even without full estrous swellings. Finally, given that females have few infants, the risks of infanticide are also plausibly lower than in the wild, although we note that chimpanzees rarely direct infanticide at group mates: males preferentially kill infants from other groups, and rare cases of infanticide by females are typically directed at new immigrants who arrive at the group with dependent offspring (Townsend et al. 2007; Wilson et al. 2014)—situations that the Ngamba chimpanzees do not experience.

Another key point concerns the structure and demography of the group. First, the group includes more females than males, although this is also common in wild communities (Emery Thompson et al. 2020; Lehmann and Boesch 2008; Mitani 2006; Nishida et al. 2003; Sugiyama 2004). We also note that our dyadic analyses examining partner sex account for this bias, and found comparable results to the individual‐focused analyses, so we have no evidence that the sex ratio impacted our reported results. However, a key difference from the wild is that African sanctuary populations do not show species‐typical patterns of male philopatry and female dispersal given that groups are composed primarily of wild‐born orphans. Since many of these individuals grew up with each other from a young age, this should reduce any sex‐biased impact of dispersal or perceived kinship on sociality. There are parallels for this possibility from the wild: for example, among wild chimpanzees at Gombe National Park in Tanzania, a population marked by unusually low female dispersal, natal females strongly prefer related social partners (Foerster et al. 2015). In the absence of kinship bonds for both sexes, any sex biases may be similarly relaxed facilitating greater similarity in male versus female social partners. One way to address this in future work would be to track the development of social bonds in such sanctuary populations as compared to the wild, to test how (lack of) dispersal impacts female versus male bonds over time. More generally, it is important to note that even in the wild chimpanzee bonds are not necessarily structured by kinship: chimpanzees can form strong, cooperative bonds with non‐kin (Bray and Gilby 2020; Langergraber et al. 2007, 2009), and indeed a majority of their bonds may be with preferred but unrelated individuals.

In conclusion, patterns of social behavior in this semi‐free‐ranging group demonstrate flexibility in the expression of sex differences in behavior in chimpanzees. As such, key sex differences that structure wild chimpanzee social behavior may be reduced or even ameliorated under relevant ecological contexts. Here, we have proposed that increases in female chimpanzee affiliation and social status may stem primarily from relaxed ecological constraints due to provisioning, which also provides a novel test of socioecological theories linking primate sociality to their feeding ecology (Wrangham 1986, 2000). More generally, this work shows that even in species with strong sex biases in behavior where males have higher dominance status and power, females can adjust their social behavior to obtain greater social support and status in some contexts. This finding is relevant to emerging theories concerning the pathways to female power in animal social groups (Davidian et al. 2022; Huchard et al. 2025). Our findings highlight the importance of intraspecific comparisons of social dynamics to disentangle which aspects of these sex differences are more fixed across populations versus more malleable, and highlight that flexible adjustments of social behavior may provide an alternative pathway to female power when conditions are favorable.

## Author Contributions

**Margaret A. H. Bryer:** writing – original draft, writing – review and editing, data curation, conceptualization. **Paige Barnes:** investigation, writing – review and editing, methodology, data curation. **Kris H. Sabbi:** conceptualization, methodology, writing – review and editing, investigation, writing – original draft. **Skylar Brodnan:** data curation, writing – review and editing. **Charlie MacKenzie:** data curation, writing – review and editing. **Isabelle Monroe:** data curation, writing – review and editing. **Natalia Camargo Peña:** data curation, writing – review and editing. **Phillip Sekulya:** methodology, investigation, writing – review and editing, project administration. **Innocent Ampeire:** methodology, investigation, writing – review and editing, project administration. **Amos Okello:** methodology, investigation, writing – review and editing. **Stanley Kyama:** methodology, investigation, writing – review and editing. **Boris Waiga:** methodology, investigation, writing – review and editing. **Hillary Aligumisiriza:** methodology, investigation, writing – review and editing. **Joseph Masereka:** methodology, investigation, writing – review and editing. **Joseph Kaale:** methodology, investigation, writing – review and editing. **Titus Mukungu:** methodology, investigation, writing – review and editing. **Joshua Rukundo:** methodology, investigation, writing – review and editing, supervision, resources, project administration. **Zarin P. Machanda:** conceptualization, investigation, funding acquisition, writing – original draft, methodology, writing – review and editing, project administration, data curation, supervision, resources, validation. **Alexandra G. Rosati:** investigation, conceptualization, funding acquisition, writing – original draft, methodology, validation, writing – review and editing, visualization, formal analysis, project administration, data curation, supervision, resources.

## Funding

This work was supported by the National Science Foundation, 1926653, 1926737; the National Institutes of Health, R37AG049395; and the Sloan Foundation, FG‐2019‐12054.

## Supporting information


**Table S1:** Predictors of observation effort. Parameters are from the model including sex (model 3), but the best‐fit model included only timeslot; reference level for the predictors are noted in the table as relevant.
**Table S2:** Predictors of rate of focal's proximity to at least one other adult. Parameters are from the model including sex (model 2), but the best‐fit model did not include sex; reference level for the predictors are noted in the table as relevant.
**Table S3:** Predictors of rate of focal's dyadic proximity. Parameters are from the full model (model 3), but the best‐fit model did not include focal's sex, partner's sex, or their interaction; reference level for the predictors are noted in the table as relevant. Note that posthoc tests revealed that focals did not show a bias for either same‐ or opposite‐sex partners.
**Table S4:** Predictors of rate of focal's total grooming (any direction). Parameters are from model 2 (including sex), but the best‐fit model did not include sex; reference level for the predictors are noted in the table as relevant.
**Table S5:** Predictors of rate of focal's dyadic total grooming (any direction). Parameters are from model 3, but the best‐fit model did not include focal's sex, partner's sex, nor their interaction; reference level for the predictors are noted in the table as relevant.
**Table S6:** Predictors of rate of focal's display rate. Parameters are from model 2 (including sex); the additional inclusion of the interaction between sex × timeslot further improved fit and revealed that males specifically were more aggressive during the first three timeslots, whereas males and females both showed little aggression in the evening timeslot.
**Table S7:** Predictors of rate of focal's directed aggression (to a victim). Parameters are from model 2 (including sex); the additional inclusion of the interaction between sex × timeslot further improved fit and revealed that males specifically were more aggressive during the first three timeslots, whereas males and females both showed little aggression in the evening timeslot.
**Table S8:** Predictors of rate of focal's dyadic directed aggression. Parameters are from model 3 including the interaction between focal's sex and victim's sex; reference level for the predictors are noted in the table as relevant.
**Table S9:** Predictors of rate of focal's coalitionary aggression. Parameters are from the model 2 (including sex) but the best‐fit model did not include sex; reference level for the predictors are noted in the table as relevant.
**Table S10:** Predictors of proportion of focal's coalitionary aggression (out all directed aggression). Parameters are from model 2, which was the best‐fit model; reference level for the predictors are noted in the table as relevant.

## Data Availability

These data are available in Dryad Digital Repository at: https://doi.org/10.5061/dryad.xd2547dx6.
