# Supplementary material for: Female Affiliation and Status in Semi‐Free‐Ranging Chimpanzees
Source: Am J Biol Anthropol. 2026 Apr 8;189(4):e70244. doi: 10.1002/ajpa.70244 (PMC13062610; doi:10.1002/ajpa.70244)
Supplement: Supplementary file 1 — Table S1: Predictors of observation effort. Parameters are from the model including sex (model 3), but the best‐fit model included only timeslot; reference level for the predictors are noted in the table as relevant. Table S2: Predictors of rate of focal's proximity to at least one other adult. Parameters are from the model including sex (model 2), but the best‐fit model did not include sex; reference level for the predictors are noted in the table as relevant. Table S3: Predictors of rate of focal's dyadic proximity. Parameters are from the full model (model 3), but the best‐fit model did not include focal's sex, partner's sex, or their interaction; reference level for the predictors are noted in the table as relevant. Note that posthoc tests revealed that focals did not show a bias for either same‐ or opposite‐sex partners. Table S4: Predictors of rate of focal's total grooming (any direction). Parameters are from model 2 (including sex), but the best‐fit model did not include sex; reference level for the predictors are noted in the table as relevant. Table S5: Predictors of rate of focal's dyadic total grooming (any direction). Parameters are from model 3, but the best‐fit model did not include focal's sex, partner's sex, nor their interaction; reference level for the predictors are noted in the table as relevant. Table S6: Predictors of rate of focal's display rate. Parameters are from model 2 (including sex); the additional inclusion of the interaction between sex × timeslot further improved fit and revealed that males specifically were more aggressive during the first three timeslots, whereas males and females both showed little aggression in the evening timeslot. Table S7: Predictors of rate of focal's directed aggression (to a victim). Parameters are from model 2 (including sex); the additional inclusion of the interaction between sex × timeslot further improved fit and revealed that males specifically were more aggressive during the first three timeslots, w [file AJPA-189-e70244-s001.docx]

**Supplemental Material**

*Female affiliation and status in semi-free-ranging chimpanzees*

**1. Interobserver reliability**

A total of 6.7% of the ten-minute focals in this dataset were reliability scans, allowing us to compare the records of two independent observers who watched the same focal individual. For the two-minute scans there was 98.1% agreement about the focal’s activity, 99.3% match for the identity of any social interaction partner, and a 96.4% match on the total number of other individuals in 1m proximity. Examining the grooming records specifically, there was agreement about the presence of grooming on 98.8% of bouts, and for those bouts where both observers recorded it there were agreement on the identity of the specific partner on 99.4% of grooming bouts and on the direction of grooming on 96.8% of bouts. Finally, there was agreement about the presence of aggression on 98.0% of events. For instances of focal aggression where both observers recorded it, there was agreement about aggressor’s aggressive behavior type (e.g., a threat versus attack) on 93.0% of events, on the victim’s identity on 100.0% of events, and on the victim’s response to aggression on 97.6% of events. Overall, this indicates we successfully collected reliable data during the study.

**2. Observation effort**

As reported in the main text, we compared observation effort for males and females across all timeslots. Table S1 shows the parameters from the third model including timeslot and focal’s sex (note that neither the inclusion of sex nor an interaction with timeslot improved model fit).

| *Predictor* | *Estimate* | *SE* | *t-value* | *p-value* |
| --- | --- | --- | --- | --- |
| Timeslot: late morning (reference: morning) | 109.844 | 4.900 | 22.418 | < 0.0001 |
| Timeslot: afternoon (reference: morning) | 106.978 | 4.900 | 21.833 | < 0.0001 |
| Timeslot: evening (reference: morning) | 136.200 | 4.900 | 27.797 | < 0.0001 |
| Focal’s Age (in years) | -0.221 | 0.610 | -0.362 | 0.719 |
| Focal’s Sex (reference = female) | 6.651 | 8.680 | 0.766 | 0.448 |

**Table S1: Predictors of observation effort.** Parameters are from the model including sex (model 3), but the best-fit model included only timeslot; reference level for the predictors are noted in the table as relevant.

**3. Proximity**

As reported in the main text, we compared proportion of scans with at least one other adult in proximity for males and females across all timeslots. Table S2 shows the parameters from the second model including timeslot and focal’s sex (note that neither the inclusion of sex nor an interaction with timeslot improved model fit).

| *Predictor* | *Estimate* | *SE* | *t-value* | *p-value* |
| --- | --- | --- | --- | --- |
| Timeslot: late morning (reference: morning) | 0.040 | 0.019 | 2.062 | 0.041 |
| Timeslot: afternoon (reference: morning) | 0.102 | 0.019 | 5.306 | < 0.0001 |
| Timeslot: evening (reference: morning) | 0.364 | 0.019 | 18.877 | < 0.0001 |
| Focal’s Age (in years) | 0.003 | 0.002 | 1.445 | 0.156 |
| Focal’s Sex (reference = female) | -0.002 | 0.026 | -0.069 | 0.946 |

**Table S2: Predictors of rate of focal’s proximity to at least one other adult.** Parameters are from the model including sex (model 2), but the best-fit model did not include sex; reference level for the predictors are noted in the table as relevant.

We then implemented several additional models as checks. First, a comparison of proportion of scans with at least one other individual in proximity collapsing across timeslot (because there was separation of data by timeslot) revealed no model improvement by including focal’s sex [χ^2^ = 0.048, df = 1, p = 0.83, n.s.]. Second, analyses of average total number of other adults in proximity revealed no improvement by including focal’s sex [χ^2^ = 0.033, df = 1, p = 0.85, n.s] nor an interaction between sex X timeslot [χ^2^ = 2.46, df = 4, p = 0.65, n.s]. Here we also accounted for number of in-view scans across models to control for observation effort, as this dependent variable was not a rate like proportion scans with at least one individual in proximity. Finally, we examined the proportion of 2-min scans at which individuals were observed ‘alone’ with no one in close proximity, and again there was no improvement by including focal’s sex [χ^2^ = 0.005, df = 1, p = 0.94, n.s] nor an interaction between sex X timeslot [χ^2^ = 4.15, df = 4, p = 0.39, n.s]. Overall, this shows no evidence for sex biases in rates of close association across males and females.

Finally, as reported in the main text, we compared dyadic association rates looking at the focal’s proximity with each other individual in the group (collapsing across all timeslots). Table S3 shows the parameters from the model including the interaction between focal’s *sex* and partner’s *sex*, although neither inclusion of sex nor the interaction improved model fit.

| *Predictor* | *Estimate* | *SE* | *t-value* | *p-value* |
| --- | --- | --- | --- | --- |
| Focal’s Age (in years) | 0.0004 | 0.0001 | 2.604 | 0.011 |
| Age difference | -0.0002 | 0.0001 | -1.899 | 0.064 |
| Focal’s sex (reference = female) | -0.0015 | 0.0014 | -1.080 | 0.285 |
| Partner’s sex (reference = female) | -0.0004 | 0.0017 | -0.259 | 0.796 |
| Focal’s sex X Partner’s sex | 0.0033 | 0.0013 | 2.442 | 0.015 |

**Table S3: Predictors of rate of focal’s dyadic proximity.** Parameters are from the full model (model 3), but the best-fit model did not include focal’ sex, partner’s sex, or their interaction; reference level for the predictors are noted in the table as relevant. Note that posthoc tests revealed that focals did not show a bias for either same- or opposite-sex partners.

**4. Grooming**

As reported in the main text, we compared proportion of scans with grooming for males and females across all timeslots. Table S4 shows the parameters from the second model including timeslot and focal’s sex (but note that neither the inclusion of focal’s sex nor the interaction with timeslot improved model fit compared to the base model).

| *Predictor* | *Estimate* | *SE* | *t-value* | *p-value* |
| --- | --- | --- | --- | --- |
| Timeslot: late morning (reference: morning) | 0.045 | 0.018 | 2.504 | 0.013 |
| Timeslot: afternoon (reference: morning) | 0.068 | 0.018 | 3.782 | < 0.0001 |
| Timeslot: evening (reference: morning) | 0.335 | 0.018 | 18.680 | < 0.0001 |
| Focal’s Age (in years) | 0.002 | 0.001 | 1.599 | 0.117 |
| Focal’s Sex (reference = female) | -0.003 | 0.018 | -0.154 | 0.878 |

**Table S4: Predictors of rate of focal’s total grooming (any direction).** Parameters are from model 2 (including sex), but the best-fit model did not include sex; reference level for the predictors are noted in the table as relevant.

We then implemented several additional models as checks. First, a comparison of proportion of scans with grooming collapsing across timeslot (because there was separation of data by timeslot) revealed no model improvement by including focal’s sex [χ^2^ = 0.02, df = 1, p = 0.89, n.s]. Second, analyses of giving grooming revealed no improvement by including focal’s sex [χ^2^ = 0.04, df = 1, p = 0.83, n.s] nor an interaction between sex X timeslot [χ^2^ = 1.26, df = 4, p = 0.87, n.s] compared to the base model. Third, analyses of receiving grooming similarly revealed no improvement by including focal’s sex [χ^2^ = 0.03, df = 1, p = 0.87, n.s] or the interaction between sex X timeslot [χ^2^ = 0.35, df = 4, p = 0.99, n.s] compared to the base model. Overall, this shows no evidence for sex biases in grooming.

As reported in the main text, we then compared dyadic grooming rates looking at the focal’s total grooming with each other individual in the group (collapsing across all timeslots). Table S5 shows the parameters from the model including the interaction between focal’s *sex* and partner’s *sex*, although neither factor nor their interaction improved model fit.

| *Predictor* | *Estimate* | *SE* | *t-value* | *p-value* |
| --- | --- | --- | --- | --- |
| Focal’s Age (in years) | 0.0001 | 0.0001 | 2.463 | 0.016 |
| Age difference | -0.0001 | 0.0000 | -1.536 | 0.132 |
| Focal’s Sex (reference = female) | -0.0002 | 0.0006 | -0.280 | 0.781 |
| Partner’s Sex (reference = female) | 0.0004 | 0.0006 | 0.625 | 0.534 |
| Focal’s Sex X Partner’s Sex | 0.0003 | 0.0007 | 0.404 | 0.686 |

**Table S5: Predictors of rate of focal’s dyadic total grooming (any direction).** Parameters are from model 3, but the best-fit model did not include focal’s sex, partner’s sex, nor their interaction; reference level for the predictors are noted in the table as relevant.

We then checked dyadic grooming based on direct (e.g., the focal giving versus receiving grooming with each other partner), following the same procedure as for total dyadic grooming. For analyses of giving grooming, neither the inclusion of focal’s sex [χ^2^ = 0.03, df = 1, p = 0.86, n.s.] or the focal’s sex X partner’s sex interaction [χ^2^ = 1.57, df = 3, p = 0.67, n.s.] improved model fit for giving grooming; these models did reveal that age difference between the focal and the partner was a significant negative predictor in addition to the positive effect of focal’s age also seen in total grooming (estimate = -0.0004, t-value = -2.32, p < 0.05 in third model). Similarly, for dyadic analyses of receiving grooming, neither the inclusion of focal’s sex [χ^2^ = 0.02, df = 1, p = 0.88, n.s.] or the focal’s sex X partner’s sex interaction [χ^2^ = 0.25, df = 3, p = 0.97, n.s.] improved model fit; here only focal’s age was a significant predictor (p < 0.05).

**5. Aggression**

As reported in the main text, we compared rates of aggression for males and females across all timeslots. Table S6 shows the parameters from the second model of analyses of displays including timeslot and focal’s sex. We also compared display rates collapsing across timeslot (as there a separation of data by timeslot) which similarly revealed that including focal’s sex improved fit [χ^2^ = 45.18, df = 1, p < 0.0001].

| *Predictor* | *Estimate* | *SE* | *t-value* | *p-value* |
| --- | --- | --- | --- | --- |
| Timeslot: late morning (reference: morning) | -0.321 | 0.066 | -4.857 | < 0.0001 |
| Timeslot: afternoon (reference: morning) | -0.417 | 0.066 | -6.303 | < 0.0001 |
| Timeslot: evening (reference: morning) | -0.491 | 0.066 | -7.410 | < 0.0001 |
| Focal’s Age (in years) | -0.005 | 0.003 | -1.510 | 0.133 |
| Focal’s Sex (reference = female) | 0.426 | 0.048 | 8.822 | < 0.0001 |

**Table S6: Predictors of rate of focal’s display rate.** Parameters are from model 2 (including sex); the additional inclusion of the interaction between *sex X timeslot* further improved fit and revealed that males specifically were more aggressive during the first three timeslots, whereas males and females both showed little aggression in the evening timeslot.

Table S7 shows the parameters from the second model of analyses of directed aggression (e.g., with a victim) including timeslot and focal’s sex. We also compared directed aggression rates collapsing across timeslot (again because of the separation of data by timeslot) which similarly revealed that including focal’s sex improved fit [χ^2^ = 44.35, df = 1, p < 0.0001].

We also broke down rates of directed aggression to examine threats and attacks separately (note that here a given bout of directed aggression might count as both a bout of threat and a bout of attack, if the focal did both behaviors over the course of the instance). On average, males engaged in threats (i.e.., behaviors such as lunges or chases) at a rate of 0.56 events per hour, whereas females did so at a rate of 0.20 per hour; all but one male were observed engaging in a threat at least once whereas 6 females were never observed to engage in a threat. The inclusion of *focal’s sex* improved model fit compared to this base model [χ^2^ = 32.24, df = 1, p < 0.000]: males engaged in more threats than females. Finally, the additional inclusion of the interaction between *focal’s sex* and *timeslot* also improved model fit compared to the second model [χ^2^ = 42.57, df = 3, p < 0.0001]; posthoc tests indicated that males showed more threats than females in the first three timeslots (p < 0.001 in all cases) but they showed similar low rates of threats in the evening.

| *Predictor* | *Estimate* | *SE* | *t-value* | *p-value* |
| --- | --- | --- | --- | --- |
| Timeslot: late morning (reference: morning) | -0.596 | 0.106 | -5.625 | < 0.0001 |
| Timeslot: afternoon (reference: morning) | -0.617 | 0.106 | -5.826 | < 0.0001 |
| Timeslot: evening (reference: morning) | -0.894 | 0.106 | -8.446 | < 0.0001 |
| Focal’s Age (in years) | -0.001 | 0.006 | -0.220 | 0.827 |
| Focal’s Sex (reference = female) | 0.596 | 0.080 | 7.492 | < 0.0001 |

**Table S7: Predictors of rate of focal’s directed aggression (to a victim).** Parameters are from model 2 (including sex); the additional inclusion of the interaction between *sex X timeslot* further improved fit and revealed that males specifically were more aggressive during the first three timeslots, whereas males and females both showed little aggression in the evening timeslot.

In terms of attacks (aggression involving physical context such as hitting, kicking, or biting), males showed a rate of 0.34 events per hour, whereas females did so at a rate of 0.19 per hour; all males were observed engaging in an attack at least once whereas 10 females were never observed to engage in an attack involving physical contact. The inclusion of *focal’s sex* improved model fit compared to this base model [χ^2^ = 30.38, df = 1, p < 0.0001]: males engaged in more attacks than females. Finally, the additional inclusion of the interaction between *focal’s sex* and *timeslot* also improved model fit compared to the second model [χ^2^ = 20.31, df = 3, p < 0.0001; note that these models were singular indicating random effects of identity approaching 0]; posthoc tests again indicated that males showed more attacks than females in the first three timeslots (p < 0.05 in all cases) but similar low rates in the evening.

Finally, Table S8 shows the parameters from the models of dyadic directed aggression also accounting for the victim’s sex, from results reported in the main text.

| *Predictor* | *Estimate* | *SE* | *t-value* | *p-value* |
| --- | --- | --- | --- | --- |
| Focal’s Age | -0.0001 | 0.00013 | -0.429 | 0.669 |
| Age difference | 0.00003 | 0.00009 | 0.302 | 0.764 |
| Focal’s sex (reference = female) | 0.0125 | 0.001 | 8.636 | < 0.0001 |
| Victim’s sex (reference = female) | 0.0001 | 0.001 | 0.089 | 0.930 |
| Focal’s sex X Victim’s sex | -0.004 | 0.002 | -2.192 | 0.029 |

**Table S8: Predictors of rate of focal’s dyadic directed aggression.** Parameters are from model 3 including the interaction between focal’s sex and victim’s sex; reference level for the predictors are noted in the table as relevant.

We also checked this result breaking down dyadic directed aggression into threats versus attacks directed at that victim (with the caveat that this dataset is then highly zero-inflated). For threats, inclusion of the aggressor’s sex again improved model fit compared to a base model [χ^2^ = 29.77, df = 1, p < 0.0001]: males aggressed more than females. Additional inclusion of the interaction between focal’s sex X victim’s sex trended to further improved fit [χ^2^ = 5.85, df = 2, p = 0.054]; as with overall directed aggression, post-hoc tests showed that males aggressed all victims more than females (p < 0.005), and males also specifically aggressed female victims more than male victims (p < 0.05). Here, the only other significant predictor in the models of dyadic threats was the age difference between the aggressor and the victim, with focals being more likely to aggress individuals who were younger than them.

For attacks, inclusion of the aggressor’s sex again improved model fit compared to a base model [χ^2^ = 44.67, df = 1, p < 0.0001]: males attacked victims more than females did. However, additional inclusion of the interaction between focal’s sex X victim’s sex did not further improve fit [χ^2^ = 1.36, df = 2, p = 0.51, n.s.]; neither age or age difference were significant predictors either. Overall, this indicates that the finding that males targeted female victims more in directed aggression is driven by an increased rate of threats directed at female victims, not attacks.

**6. Coalitions**

For coalitions, following reporting from the main text Table S9 shows the parameters from the models of coalition rates (e.g., rate of coalitionary aggression across all time), whereas Table S10 shows analyses of coalition proportion (e.g., proportion of directed aggression that involved coalitions, to account for the different rates at which males versus females engage in directed aggression overall).

| *Predictor* | *Estimate* | *SE* | *t-value* | *p-value* |
| --- | --- | --- | --- | --- |
| Focal’s Age | 0.000 | 0.001 | 0.646 | 0.522 |
| Focal’s Sex (reference = female) | 0.012 | 0.010 | 1.314 | 0.196 |

**Table S9: Predictors of rate of focal’s coalitionary aggression.** Parameters are from the model 2 (including sex) but the best-fit model did not include sex; reference level for the predictors are noted in the table as relevant.

| *Predictor* | *Estimate* | *SE* | *t-value* | *p-value* |
| --- | --- | --- | --- | --- |
| Focal’s Age | 0.002 | 0.005 | 0.391 | 0.698 |
| Focal’s Sex (reference = female) | -0.163 | 0.068 | -2.400 | 0.021 |

**Table S10: Predictors of proportion of focal’s coalitionary aggression (out all directed aggression).** Parameters are from model 2, which was the best-fit model; reference level for the predictors are noted in the table as relevant.
